# US Emergency Department Use and Operations Amid Natural Disasters: A Narrative Review

**DOI:** 10.5811/westjem.49118

**Published:** 2026-02-22

**Authors:** Atrik Patel, Ashley A. Foster, Erica Y. Popovsky, Andrea Fawcett, Jennifer A. Hoffmann

**Affiliations:** * Northwestern University Feinberg School of Medicine, Chicago, Illinois; † University of California, San Francisco, Department of Emergency Medicine, San Francisco, California; ‡ Northwestern University Feinberg School of Medicine, Ann & Robert H. Lurie Children’s Hospital of Chicago, Division of Emergency Medicine, Department of Pediatrics, Chicago, Illinois; § Ann & Robert H. Lurie Children’s Hospital of Chicago, Department of Clinical and Organizational Development, Chicago, Illinois

## Abstract

In the United States from 2014–2024, an average of 18.2 national disasters per year caused over a billion dollars in inflation-adjusted damage, compared with 3.3 national disasters per year during the 1980s. The increased frequency and intensity of severe weather phenomena—attributed by climate science experts to climate change—have raised concerns about national emergency preparedness. One aspect of emergency preparedness is the functioning of emergency departments (ED). In this narrative review, we examine patterns of ED use and operations amid natural disasters in the US, with a special focus on vulnerable populations. The review highlights studies comparing ED use patterns between periods of disaster and non-disaster for specific disaster types, including hurricanes, wildfires, floods, winter storms, and earthquakes, as well as studies that identify disaster-mediated changes in ED visits among specific populations, including the elderly, individuals experiencing homelessness, children and youth with special health care needs, and individuals with chronic medical and psychiatric conditions. Finally, we highlight the challenges posed to EDs by these disasters, including crowding, resource scarcity, and operational strain, and proposed steps to strengthen ED preparedness for climate-related disasters.

## INTRODUCTION

When reflecting on major challenges posed to communities in the 21^st^ century, climate change stands out as a significant existential concern.^1^ The Intergovernmental Panel on Climate Change Sixth Assessment Report found that worsening climate conditions have increased the frequency and intensity of many weather- and climate-related extremes worldwide.^2^ Specifically in the United States, the number of natural disasters causing over a billion dollars in inflation-adjusted damage has averaged 18.2 per year between 2014–2024, as compared to the average of 3.3 per year during the 1980s.^3^ The devastating impacts of consecutive occurrences of natural disasters on a nation’s economic and social well-being have led to advocacy for more attention to be placed on disaster risk management.^4^ Following these extreme weather events, questions have arisen regarding societal readiness to mitigate the damaging effects of disasters on human health. In the US, recent trends in emergency responses have revealed concerning metrics related to the nation’s ability to handle immediate, crisis-based health challenges posed by similar surge events. During the COVID-19 pandemic, for example, many emergency departments (ED) reported issues of crowding and resource shortages that strained their ability to provide high-quality patient care.^5^

Given these challenges to emergency health preparedness and infrastructure due to climate-related disasters, our goal was to examine current patterns of ED utilization and operations in the US during natural disasters. In this review we discuss these patterns, stratified by disaster type, with particular attention to the types and timing of ED presentation that were directly linked to these natural disasters or resulted in exacerbation of existing health conditions. We describe specific population groups that may face increased susceptibility to health-related challenges requiring emergency attention due to these disasters. Additionally, we consider gaps in emergency health preparedness and response that may impact disaster management and identify steps that can be taken to fill these gaps.

## METHODS

We developed the literature search in consultation with a medical librarian (AF). Searches were performed in the following databases: PubMed; Google Scholar; Cochrane Library; CINAHL; Embase; and PsycINFO. Search queries used combinations of keywords (including “emergency department,” “climate change,” “natural disaster,” “extreme weather,” “health care delivery,” and “operations”), as well as controlled vocabulary terms. Searches were restricted to studies published in English and were not limited by publication date. For each study identified, key information was extracted, including disaster type, study characteristics, affected populations, and ED outcomes. Data extraction and inclusion decisions were conducted by the lead author, who was aware of the project hypothesis. We included studies based on relevance to US ED utilization or operations in the context of natural disasters, rather than according to pre-specified inclusion or exclusion criteria.

## RESULTS

### Natural Disasters

#### Hurricanes

Due to the impact of climate change, the severity of US hurricanes has increased over the past three decades,^6^ raising risks of injury. In 29 counties in Eastern North Carolina, the week following Hurricane Irene (2011) saw 2,252 injury-related ED visits, a 22.3% increase from a 2010 reference week.^7^ In New Jersey, ED visits for tree-related injuries increased in the three months after Superstorm Sandy (2012) when compared to the same period during the prior year (incidence rate ratio [IRR] 1.67; 95% CI, 1.13–2.47) and subsequent year (IRR 2.47; 95% CI, 1.62–3.78).^8^ In the two days after the 1989 landfall of Hurricane Hugo, a daily average of 1,146 ED visits was noted in several inland North Carolina counties, a rise from the averages of two weeks prior (821 visits) and 3–14 days after (833 visits).^9^ Of the visit totals specific to hurricane-related concerns (comprising 30% of total ED patients in the two days after the hurricane and 10% of total patients across the rest of the study period), 88% involved injuries, with nearly half of the total disaster-related cases stemming from insect stings and wounds.^9^

Increases in the number of injuries amid hurricanes, however, are not the only impacts noted. In Long Island, a 4% increase in respiratory disease-related ED visits occurred in the days after Superstorm Sandy (October 30–November 1, 2012) when compared to preceding days (October 1–October 28) (*P* < .001).^10^ At a South Florida children’s hospital, ED visits increased 3.2% for gastroenteritis and 0.9% for cellulitis in the first and second weeks after Hurricane Andrew (1992), respectively, when compared to the week before (*P* < .05).^11^ Among New York City adults ≥ 65 years of age, EDs noted visit increases during the first week after Superstorm Sandy for cardiovascular disease (relative risk [RR] 1.10; 95% CI, 1.02–1.19); injuries and poisoning (RR 1.19, 95% CI, 1.10–1.28); renal disease (RR 1.44; 95% CI, 1.22–1.72); respiratory disease (RR 1.35; 95% CI, 1.21–1.49); and skin and soft tissue infections (RR 1.20; 95% CI, 1.03–1.39) compared to all other days between 2005–2014.^12^

Patterns of hurricane-related ED use reflect spatial differences. In 344 US counties affected by seven hurricanes (2005–2016), the magnitude of changes identified in population rates of weekly, post-hurricane ED visits by age and disease category were generally larger for counties in closer geographic proximity to the hurricane path.^13^ Furthermore, temporal differences in ED use patterns are noted. Compared to pre- and post-hurricane data, studies have found decreased ED use during a hurricane, likely due to individuals sheltering in place.^10^,^14^ In coastal Southeastern Virginia, six EDs experienced a 46% decline in daily visit volume on the day of Hurricane Isabel’s 2003 landfall compared to the prior six months’ daily average.^15^ A consequence of sheltering in place, however, is a rapid influx of ED visits immediately post-hurricane. Notably, in the five days after Hurricane Isabel’s landfall, the daily visit volume in the six studied EDs increased by 25% when compared to the same pre-hurricane daily average.^15^

#### Wildfires

Climate change has intensified US wildfire seasons, which pose distinct health concerns.^16^ Unlike hurricanes, which elevate ED visits for injuries and other medical issues,^7^–^12^ wildfires mainly increase ED visits for respiratory and cardiovascular concerns due to smoke exposure, particularly among those with pre-existing conditions, individuals < 5 years of age, and individuals > 65 years of age.^17^ Visits to the ED for a range of respiratory conditions increase following wildfire smoke exposure.

In June 2008, after wildfires in Eastern North Carolina, the number of ED visits rose for acute bronchitis and pneumonia (cumulative RR 1.59; 95% CI, 1.07–2.34), asthma (cumulative RR 1.65; 95% CI, 1.25–2.10), and chronic obstructive pulmonary disease (COPD) (cumulative RR 1.73; 95% CI, 1.06–2.83) in affected counties compared to referent counties.^18^ Because wildfire smoke can travel long distances, it represents a broad-reaching health hazard. The 2023 Canadian wildfires, for instance, led to increases in ED visits for chief complaints related to asthma, COPD, or wheezing in New York City during the resulting smoke wave (June 6–8) when compared to adjacent reference days (IRR 1.44; 95% CI, 1.31–1.58).^19^

Studies analyzing ED usage patterns demonstrate that wildfire smoke most severely affects young children’s respiratory health.^20^,^21^ In Southern California, during the days of the 2007 wildfires, ED visits rose for dyspnea (3.2 excess visits per day; *P* = .01) and asthma (1.5 excess visits per day; *P* = .02) compared to two weeks prior.^22^ These fires, however, were particularly detrimental to the respiratory health of children 0–4 years of age, who presented to EDs for respiratory diagnoses at a higher rate during the peak exposure period (October 22–26) when compared to prior reference days (RR 1.70; 95% CI, 1.32–2.19).^20^ Similarly, during the 2017 Lilac Fire in San Diego, children 0–5 years of age had 7.3 excess daily respiratory ED visits during the fire compared to reference days from the prior year (95% CI, 3.0–11.7).^21^

In contrast to respiratory conditions, ED visits for cardiovascular complaints increase predominantly among the elderly,^23^ with delayed presentations.^24^ In California, wildfire smoke exposure in 2015 increased ED visits for cardiovascular diagnoses among individuals ≥ 65 years of age when compared to reference days without smoke (RR 1.15; 95% CI, 1.09–1.22).^23^ Furthermore, while respiratory complaints presented more immediately to EDs after California wildfire smoke events between 2016–2019, cardiovascular complications were delayed in presentation by several days.^2^ Wildfires may also alter care-seeking behavior for non-cardiorespiratory concerns. In California, when comparing non-federal hospital ED visits to wildfire smoke concentration estimates between 2006–2017, ED visit rates were estimated to decline for accidental injuries by 19% on high-smoke intensity days when compared to smoke-free days within the same ZIP code (95% CI, 9–30%).^25^ A likely contributor to this decrease may have been avoidance of care for non-urgent concerns amid poor air quality.^25^

#### Floods, Winter Storms, and Earthquakes

While fewer studies have examined impacts on US EDs from floods, winter storms, and earthquakes, these events may also affect ED use patterns. In Massachusetts between 2003–2007, analysis of ED and outpatient visits found that males had an increased risk for *Clostridium difficile* infection in the 7-to-13 days after coastal and flash flood events compared to reference days from four weeks before and/or after the events (odds ratio [OR] 3.21; 95% CI, 1.01–10.19).^26^ Flood events due to heavy precipitation have been associated with ED visit increases in affected ZIP code tabulation areas (ZCTA), as noted during the 2019 Texas flood events, which led to a 37% increase in dehydration-related visits among children 0–5 years of age (95% CI, 8%–73%) and a 45% increase among children 6–17 years of age (95% CI, 9%–92%) compared to control ZCTAs.^27^ Study of flood-exposed ZCTAs in the continental US from 2008–2017 revealed increases in number of ED visits for metabolic and kidney conditions (IRR 1.08; 95% CI, 1.06–1.11) and injuries (IRR 1.05; CI, 1.04–1.06) among individuals ≥ 65 years of age when compared to ED visits occurring four weeks prior to the events.^28^

After winter storms, an increase in ED visits for carbon monoxide (CO) poisoning has been observed. During the 2009 Kentucky ice storm, 202 ED visits for CO poisoning were noted, a nearly 18-fold increase from the 11 ED visits noted during the same period in the prior year.^29^ Increased risk for CO poisoning likely stems from exposure sources such as portable generators used during storm-related power outages.^29^,^30^ Additional concerns include potential ED visits for fall-related injuries from icy conditions,^31^ as well as injuries sustained from storm-associated damage.^32^

Earthquakes can pose challenges to EDs due to their destructive nature and unpredictable timing. Immediately following the 1994 Northridge earthquake in Southern California, ED visits to Northridge Hospital tripled (343 visits) relative to prior average daily patient volumes (110 visits), with chief presenting complaints including lacerations, contusions, and obstetric/gynecological health concerns.^33^ Following the 1989 Loma Prieta earthquake in Northern California, 51 hospitals in six affected counties logged 12,407 ED visits over three days, a 15% increase compared to the prior week’s baseline ED census.^34^ Common presenting injuries included contusions, fractures, and open wounds (Table 1).^34^

### Vulnerable Population

#### Elderly

The elderly face disproportionate impacts from natural disasters, reflected in higher ED use. In the month following Superstorm Sandy in New Jersey, ED cases for both physical and mental health concerns for patients ≥ 65 years of age surged compared to the same reference month two years before and one year after (*P* < .001).^35^ Notably, trauma-related injuries showed the greatest rise (+ 880 cases), followed by mental illness (+ 169 cases).^35^ Similarly, during the three weeks after Superstorm Sandy in New York City, ED use increased by 2% in patients ≥ 85 years of age when compared to prior reference weeks (*P* < .01), with increased presentations of dementia (+ 1.7%), homelessness (+ 2.2%), general non-specific symptoms (+ 1.7%), and malnutrition (+ 1.3%).^36^ In Lower Manhattan, Superstorm Sandy resulted in a 114% increase in ED use by patients ≥ 80 years of age immediately post-disaster when compared to a pre-storm baseline visit average (*P* < .01), with increased presentations for dialysis, respiratory device failure, and social causes such as needing food and water (*P* < .05).^37^ After Hurricane Irma (2017), assisted living residents in Florida ≥ 65 years of age who evacuated had higher odds of ED visitation at the 30-day mark post-disaster when compared to a reference group of residents who sheltered in place (adjusted OR 1.16; 95% CI, 1.01–1.33).^38^ This finding raises further questions regarding the vulnerability of the elderly population when considering emergency preparedness and response.

#### Individuals Experiencing Homelessness

Visits to the ED by individuals experiencing homelessness also rise after severe weather events. In the week following Superstorm Sandy, New York City saw an increase in ED visits for primary and secondary diagnoses of “inadequate housing” or “lack of housing” compared to a prior baseline weekly average (*P* < .01).^39^ Visits for primary diagnoses of “inadequate housing” increased 60-fold (0.2 to 12 visits), while secondary diagnoses increased nearly 40-fold (2.1 to 83 visits).^38^ Visits for primary diagnoses of “lack of housing” increased approximately 6-fold (2.6 to 15 visits), while secondary diagnoses increased just over 1-fold (261.1 to 289 visits).^39^ These increases likely reflected both pre-existing conditions of inadequate housing or homelessness and new housing insecurity or loss due to the disaster.^39^

#### Children and Youth with Special Healthcare Needs

Across disasters, children and youth with special healthcare needs experience increased health challenges. In the months following Hurricane Katrina in 2005, children with chronic conditions seen in the ED, ambulatory care center, and other healthcare facilities in the New Orleans metropolitan area were more likely than children without these conditions to experience worsening asthma (16.3% vs 1.9%, *P* < .001), run out of medications (33.9% vs 7.9%, *P* < .001), and experience at the minimum one disruption in their care (58.4% vs 38.3%, *P* < .001).^40^ During disasters, children and youth with special healthcare needs can face issues accessing vital resources, such as medications^40^ and electrical power for life-sustaining equipment.^41^ In non-disaster conditions, these children and youth use EDs at a disproportionately higher rate than children without special healthcare needs, with 25.3% of this cohort visiting the ED at least one time over 12 months compared to 14.5% of children without special healthcare needs.^42^ Consequently, when emergency resources are strained during disasters, children and youth with special healthcare needs face additional vulnerability.^41^

#### Individuals with Chronic Health Conditions

A variety of chronic health conditions may lead individuals to visit the ED because of disaster-related disruptions to care. In the weeks following Hurricane Katrina, 58% of the 21,673 total visits across 29 emergency treatment facilities in New Orleans were for illness (12,567 visits).^43^ Of this proportion of illness-related visits, 24.3% were attributable to chronic disease and related conditions (3,054 visits), among them 1,001 visits for cardiovascular disease, 371 visits for chronic lower-respiratory disease, and 294 visits for obstetric/gynecological conditions^43^

Superstorm Sandy posed threats to the health and medical needs of patients with diabetes. In the week following the storm, New York City patients with a secondary diagnosis of diabetes visited the ED in increased numbers for chronic bronchitis (+ 8 weekly cases), hypertension (+ 11 weekly cases), hypertensive kidney disease (+ 8 weekly cases), and myocardial infarction (+ 8 weekly cases) when compared to a prior weekly ED visit baseline.^44^ In New Jersey, an 84% increase in ED visits was noted among patients with a primary diagnosis of type II diabetes seeking acute care during the week of Superstorm Sandy when compared to a reference week from the year before (95% CI, 1.12–3.04; *P* = .01).^45^ Increased ED use by individuals with chronic conditions can also occur in the long term following natural disasters. Medicare enrollees with diabetes in Louisiana, Mississippi, Texas, and Alabama impacted by Hurricanes Katrina and Rita in 2005 had an additional 21,583 ED visits three years following the hurricanes when compared to a control group not impacted by the disasters (95% CI, 11,676 – 31,490).^46^

#### Individuals with Mental Health Symptoms

Beyond immediate physical health concerns, individuals may also experience or face further exacerbation of mental health challenges amid natural disasters. When compared to the year prior to Superstorm Sandy, in the year after, psychiatric ED visits at Maimonides Medical Center in New York City increased by close to 32% (*P* < .001) (Table 2).^47^ Other operational New York City hospitals noted similar persisting surges in psychiatric ED visits after the storm, likely due to displaced patients from non-operational facilities.^48^ The consequence of these surges in visits may be longer ED boarding times for individuals seeking inpatient psychiatric care, as noted in EDs across Louisiana after Hurricane Katrina.^49^ In the months following this hurricane, challenges in mental health service availability were noted in New Orleans, including drops in pre-hurricane totals of practicing psychiatrists (208 to 42) and available mental health beds (487 to 190).^50^ Resulting strain placed on EDs that were boarding psychiatric patients is likely in part reflected in reported hospital compensation rates, with a New Orleans hospital noting a greater than 140% hurricane-related increase in uncompensated care ($17 million to $41 million), 90% of which was attributable to its ED.^50^

## DISCUSSION

### Emergency Care Preparedness and Future Steps

#### Current Landscape of Emergency Response to Natural Disasters

Natural disasters result in increased strain on emergency healthcare systems nationwide, with high-risk populations such as the elderly, individuals experiencing homelessness, and individuals who may require ongoing medical attention being disproportionately impacted.^35^–^41^,^43^–^50^ This underscores the importance of ED preparedness strategies to anticipate patient surges without shifting to contingency or crisis standards of care. During natural disasters, EDs act as safety nets to address unmet healthcare needs while simultaneously providing treatment for emergent health conditions.^51^ Consequent crowding and patient surges, however, may constrain the ED’s ability to meet both needs.^52^,^53^ In response to scarcities in space, staffing, and resources, EDs may, therefore, transition from conventional operating conditions to operating under contingency or crisis standards of care.^54^

Issues of crowding are further compounded when disaster-related hospital closures lead to operational challenges for EDs that remain open. During Tropical Storm Allison (2001), severe flooding led to the closure or service curtailment of nine Houston hospitals, reducing capacity by close to 1,700 beds.^55^ As a result, wait times increased to 18–21 hours in EDs still functioning.^55^ In this manner, hospital closures may compel individuals to seek care at EDs in place of their typical sources of medical care.^56^ However, as EDs may not have access to the patients’ prior medical records,^57^ this may present barriers to their continuity of care. Given that natural disasters exacerbate challenges, they may also affect the ability of ED staff to present to work due to competing obligations, such as addressing the immediate needs of their dependents.^58^ Even among staff able to be present, anxieties surrounding their service may remain, including concerns about their own personal safety and the capacity of the workplace to meet their basic human needs.^59^

Challenges to ED resources imposed by natural disasters may further extend beyond the period during and immediately after these disasters. Disruptions to care and impacts to operations may be prolonged, as noted for EDs in New Orleans after Hurricanes Ida (2021) and Katrina (2005), which faced significant challenges addressing personnel recall and vacancies after the disasters.^60^,^61^ The ED staff who serve during and immediately after a disaster may experience extended personal challenges as well, as noted among ED nurses in New Orleans who faced longitudinal symptoms of post-traumatic stress disorder after responding to Hurricane Katrina.^62^

Consideration must additionally be given to broader national discourse and directives that may impact emergency care efforts. For instance, the federal government has signaled intentions to downsize or dismantle the Federal Emergency Management Agency (FEMA),^63^ which could have implications for EDs in disaster scenarios. While FEMA does not directly manage disasters, it supports and coordinates federal resources to local systems upon a state’s request to bolster local response capacity. Although the scope and implementation of this proposed downsizing remain uncertain, if enacted these changes could increase the operational burden on local systems by limiting their access to critical federal assistance, potentially exacerbating existing strain on EDs during disasters.

#### Future Steps to Improve Emergency Care Delivery During and After Natural Disasters

As the frequency and severity of natural disasters in the United States increase, ED disaster preparedness will be crucial. Emergency planners can use ED surveillance data to understand how previous natural disasters impacted regional EDs to prepare for future severe weather events.^64^ Specific attention should further be given to creating syndromic surveillance tools that consider the impacts of natural disasters on ED visits related to mental health and psychological trauma.^65^ Additionally, clinical frameworks that outline how to efficiently and effectively assess and manage climate- and disaster-related health challenges can be created and distributed to ED teams.^66^

To better address crowding, local government and regional healthcare coalitions can make more non-ED locations available to the public and inform them about where they can receive medical treatment at these locations during disasters. For example, offering non-urgent medical screening and treatment in evacuee evaluation centers can reduce strain on local EDs.^52^ Official messaging regarding the utility of these spaces can be relayed to the public through social media, emergency calls, and other mechanisms of contact. In conjunction, telehealth models can be developed to connect emergency medical services (EMS) with ED staff to enhance on-scene patient assessment and intervention,^67^ thereby improving triage and potentially limiting unnecessary ED transports. Adequate funding to support EMS and train EMS personnel to respond to disasters is critical to the success of this model.

A well-coordinated disaster response is not limited to immediate emergency response needs; it also addresses the long-term consequences of natural disasters. To rebuild emergency care capacity after disasters, for example, medical operations coordination centers can be formed to monitor patient volumes and balance the load across functioning EDs by directing patient transports based on resource availability.^68^ To support ED staff preparedness, ED disaster planners must account for personal concerns and staff needs that may impede their ability to respond to a disaster.^58^ Accordingly, emergency planning should clearly delineate the expected duties of ED staff during natural disasters, while also addressing personal or professional obstacles for staff to engage in their work.^69^ Furthermore, hospital administrators should offer ED staff short-term and long-term treatment options for emotional difficulties faced while handling health crises caused by natural disasters.^62^

Ultimately, the responsibility for ensuring that emergency health services can meet increased demands during and after natural disasters rests not solely on ED stakeholders but also on broader health systems, public health, and public service representatives. A concerted effort must be made by all groups in tandem to address the impacts of the disaster cycle through leadership in public policy and intervention,^70^ further research and education,^70^ and advocacy for greater investment in disaster readiness. Such operational, political, and academic considerations should both bolster emergency preparedness and address explicit contributors to the noted rise in the force and frequency of natural disasters, namely the worsening effects of climate change.^71^

## LIMITATIONS

This narrative review has several limitations. The studies were heterogeneous in design, data source, and regional focus, limiting direct comparisons. Many studies further relied on matched cohorts or retrospective designs, which may not have accounted for all confounding variables and could not rule out multifactorial influences on observed outcomes. Data on certain disasters, such as floods, winter storms, and earthquakes, as well as vulnerable populations, including rural or uninsured patients, remain sparse. Access to standardized patient-level and operational data, such as ED visit timing, acuity, resource use, and staffing, would have enhanced the discussion by enabling more detailed comparisons and clearer identification of factors associated with ED surges and care disruptions. Future multicenter, prospective, and mixed-methods studies could clarify these patterns while capturing insights from both patients and clinicians.

## CONCLUSION

As critical access points to care, emergency departments serve as a cornerstone of disaster response and recovery. Amid the growing frequency and severity of climate-related natural disasters, it is imperative that EDs take steps to ensure they remain well-positioned to deliver operationally efficient care to meet community needs. Notably, climate-based challenges can compromise health equity. As has been detailed, a large body of data shows that the most socially and medically vulnerable are especially likely to suffer adverse outcomes and face barriers to healthcare during natural disasters. Consequently, in the face of climate change, health justice must remain the guiding compass for strengthening ED resilience and emergency care preparedness.

## Figures and Tables

**Table 1:** Summary of studies related to impacts of natural disasters on emergency department volumes in the United States.

Study description	Population	Outcomes and Effect Size
**Hurricanes**		
Eastern North Carolina, Hurricane Irene (2011)^7^	All ED patients	Increase in injury-related ED visits (one week after disaster): + 22.3% (reference: same week from year before disaster)
New Jersey, Superstorm Sandy (2012)^8^	All ED patients	Increase in tree injury-related ED visits (three months after disaster): IRR 1.67; 95% CI, 1.13–2.47 (reference: same three months from year before disaster) IRR 2.47; 95% CI, 1.62–3.78 (reference: same three months from year after disaster)
North Carolina, Hurricane Hugo (1989)^9^	All ED patients	Increase in average daily ED visits (two days after disaster): + 325 visits (reference: two weeks before disaster) + 313 visits (reference: 3-14 days after disaster) Large proportion of ED visits related to disaster, particularly for injuries: Disaster-related visits = 30% of total ED volume (two days after disaster) Disaster-related visits = 10% of total ED volume (across rest of 2-week study period) 88% of disaster-related visits = injuries Approximately 50% of disaster-related visits = insect stings and wounds
Long Island, Superstorm Sandy (2012)^10^	All ED patients	Increase in respiratory disease-related ED visits (three days after disaster): + 4%; *P* < .001 (reference: 28 days before disaster)
South Florida, Hurricane Andrew (1992)^11^	Pediatric ED patients	Increase in gastroenteritis-related ED visits (one week after disaster): + 3.2%; *P* < .05 (reference: one week before disaster) Increase in cellulitis-related ED visits (two weeks after disaster): + 0.9%; *P* < .05 (reference: one week before disaster)
New York City, Superstorm Sandy (2012)^12^	ED patients ≥ 65 years	Increase in cardiovascular disease-related ED visits (one week after disaster): RR 1.10; 95% CI: 1.02–1.19 (reference: all other days between 2005–2014) Increase in injuries and poisoning-related ED visits (one week after disaster): RR 1.19; 95% CI, 1.10–1.28 (reference: all other days between 2005–2014) Increase in renal disease-related ED visits (one week after disaster): RR 1.44; 95% CI, 1.22–1.72 (reference: all other days between 2005–2014) Increase in respiratory disease-related ED visits (one week after disaster): RR 1.35; 95% CI, 1.21–1.49 (reference: all other days between 2005–2014) Increase in skin and soft tissue infection-related ED visits (one week after disaster): RR 1.10; 95% CI, 1.02–1.19 (reference: all other days between 2005–2014)
344 US counties, seven hurricanes (2005–2016)^13^	All ED patients	Larger magnitude of changes in population rates of weekly ED visits for counties closer to path of hurricane (post-hurricane, by age and disease category)
Coastal Southeastern Virginia, Hurricane Isabel (2003)^15^	All ED patients	Decrease in daily ED visit volume (day of disaster): - 46% (reference: six months before disaster) Increase in daily average visit volume (five days after disaster): + 25% (reference: six months before disaster)
**Wildfires**		
Eastern North Carolina, 2008 wildfires^18^	All ED patients	Increase in ED visits for acute bronchitis and pneumonia (affected counties): Cumulative RR 1.59; 95% CI, 1.07–2.34 (reference: control counties) Increase in ED visits for asthma (affected counties): Cumulative RR 1.65; 95% CI, 1.25–2.10 (reference: control counties) Increase in ED visits for chronic obstructive pulmonary disease (affected counties): Cumulative RR 1.73; 95% CI, 1.06–2.83 (reference: control counties)
New York City, 2023 Canadian wildfires^19^	All ED patients	Increase in ED visits for asthma, chronic obstructive pulmonary disease, or wheezing (days of smoke wave): IRR 1.44; 95% CI, 1.31–1.58 (reference: adjacent, non-smoke days)
Southern California, 2007 wildfires^22^	All ED patients	Increase in daily ED visits for dyspnea (period of disaster): + 3.2 excess visits; *P* = .01 (reference: two weeks before disaster) Increase in daily ED visits for asthma (period of disaster): + 1.5 excess visits; *P* = .02 (reference: two weeks prior to disaster)
Southern California, 2007 wildfires^20^	ED patients 0–4 years of age	Increase in ED presentations for respiratory diagnoses (period of highest exposure): IRR 1.70; 95% CI, 1.32–2.19 (reference: control days before the disaster)
San Diego, 2017 Lilac Fire^21^	ED patients 0–5 years of age	Increase in daily ED visits for respiratory concerns (period of disaster): + 7.3 excess visits (reference: control days from year before disaster)
California, 2015 wildfire smoke events^23^	ED patients ≥ 65 years	Increase in ED visits for cardiovascular diagnoses (periods of wildfire smoke exposure): RR 1.15; 95% CI, 1.09–1.22 (reference: days without smoke)
California, 2016–2019 wildfire smoke events^24^	All ED patients	Delay in ED presentation of cardiovascular complications (periods of wildfire smoke exposure): Several days (reference: timing of respiratory complaint presentation)
California, 2006–2017 wildfire smoke concentration estimates^25^	All ED patients	Decrease in estimated ED visit rates for accidental injuries (days with high smoke intensities): - 19%; 95% CI, 9–30% (reference: days without smoke)
**Floods**		
Massachusetts, 2003–2007 coastal and flash flood events^26^	Male ED and outpatient patients	Increase in risk for *Clostridium difficile* infection (7–13 days after disaster): Odds ratio 3.21; 95% CI, 1.01–10.19 (reference: control days from four weeks before and/or after disaster)
Texas, 2019 flood events^27^	Pediatric ED patients 0–17 years of age	Increase in dehydration-related ED visits among patients 0–5 years of age (affected ZCTAs): + 37%; 95% CI, 8–73% (reference: control ZCTAs) Increase in dehydration-related ED visits among patients 6–17 years of age (affected ZCTAs): 45%; 95% CI, 9–92% (reference: control ZCTAs)
Continental US, 2008–2017 flood events^28^	ED patients ≥ 65 years of age	Increase in metabolic and kidney conditions-related ED visits (affected ZCTAs): IRR 1.08; 95% CI, 1.06–1.11 (reference: four weeks before disaster) Increase in injury-related ED visits (affected ZCTAs): IRR 1.05; 95% CI, 1.04–1.06 (reference: four weeks before disaster)
**Winter Storms**		
Kentucky, 2009 ice storm^29^	All ED patients	Increase in carbon monoxide poisoning-related ED visits (period of disaster): + 18-fold (reference: control period from year before)
**Earthquakes**		
California, Northridge earthquake (1994)^33^	All ED patients	Increase in total ED visits (immediately following disaster): + 3-fold (reference: average daily patient volumes before disaster) Chief presenting complaints = lacerations, contusions, and obstetric/gynecological health concerns
California, Loma Prieta earthquake (1989)^34^	All ED patients	Increase in total ED visits (day of disaster and two days after disaster): + 15% (reference: baseline ED census from week before disaster) Common presenting injuries = contusions, fractures, and open wounds

*ED*, emergency department; *IRR*, incident rate ratio; *RR*, risk ratio; *ZCTA*, ZIP code tabulation area.

**Table 2:** Summary of studies related to impacts of natural disasters on vulnerable populations and their use of emergency departments in the United States.

Study description	Population	Outcomes and Effect Size
**Elderly populations**		
New Jersey, Superstorm Sandy (2012)^35^	ED patients ≥ 65 years of age	Surge in both physical and mental health concern-related ED cases (one month after disaster): *P* < .001 (reference: averages of control months two years before and one year after disaster) Largest increase in ED cases related to trauma-related injuries: + 880 cases Second largest increase in ED cases related to mental illness: + 169 cases
New York City, Superstorm Sandy (2012)^36^	ED patients ≥ 85 years of age	Increase in overall ED utilization (three weeks after disaster): + 2%; *P* < .01 (reference: control weeks prior to disaster) Increase in dementia-related ED visits: +. 1.7% Increase in homelessness-related ED visits: + 2.2% Increase in general nonspecific symptoms-related ED visits: + 1.7% Increase in malnutrition-related ED visits: + 1.3%
Lower Manhattan, Superstorm Sandy (2012)^37^	ED patients ≥ 80 years of age	Increase in overall ED utilization (immediately after disaster): + 114%; *P* < .01 (reference: baseline ED visit average before disaster) Increase in ED presentations for dialysis, respiratory device failure, and social causes (needing food and water): *P* < .05
Florida, Hurricane Irma (2017)^38^	Evacuated assisted living residents ≥ 65 years of age	Increase in odds of ED visitation (30 days after disaster): Adjusted odds ratio 1.16; 95% CI, 1.01–1.33 (reference: sheltered assisted living residents)
**Individuals Experiencing Homelessness**		
New York City, Superstorm Sandy (2012)^39^	All ED patients	Increase in ED visits for primary and secondary diagnoses of “inadequate housing” or “lack of housing” (one week after disaster): *P* < .01 (reference: baseline weekly average before disaster) Increase in primary diagnoses of “inadequate housing”: + 60-fold Increase in secondary diagnoses of “inadequate housing”: Nearly + 40-fold Increase in primary diagnoses of “lack of housing”: Approximately + 6-fold Increase in secondary diagnoses of “lack of housing”: Just over + 1-fold
**Children and Youth with Special Healthcare Needs**		
New Orleans, Hurricane Katrina (2005)^40^	Pediatric patients seen in the ED, ambulatory care center, and other healthcare facilities	Greater likelihood of experiencing worsening asthma (months after disaster): 16.3% vs 1.9%; *P* < .001 (reference: percentage of patients without special healthcare needs experiencing same) Greater likelihood of running out of medications (months after disaster): 33.9% vs 7.9%; *P* < .001 (reference: percentage of patients without special healthcare needs experiencing same) Greater likelihood of experiencing at the minimum one disruption in their care (months after disaster): 58.4% vs 38.3%; *P* < .001 (reference: percentage of patients without special healthcare needs experiencing same)
**Individuals with Chronic Health Conditions**		
New Orleans, Hurricane Katrina (2005)^43^	All emergency treatment facilities patients	Large proportion of emergency visits for illness (weeks after disaster): 58%, 12,567 visits (reference: total emergency visits, 21,673 visits) Large proportion of illness-related visits for chronic disease and related conditions: 24.3%, 2,054 visits (reference: total illness-related visits, 12,567 visits) Common condition = cardiovascular disease: 1,001 visits (reference: chronic disease and related conditions-based visits, 2,054 visits) Common condition = chronic lower-respiratory disease: 371 visits (reference: chronic disease and related conditions-based visits, 2,054 visits) Common condition = obstetric/gynecological conditions: 294 visits (reference: chronic disease and related conditions-based visits, 2,054 visits)
New York City, Superstorm Sandy (2012)^44^	ED patients with secondary diagnosis of diabetes	Increase in weekly, chronic bronchitis-related visits (one week after disaster): + 8 cases (reference: weekly baseline visits before disaster) Increase in weekly, hypertension-related visits (one week after disaster): + 11 cases (reference: weekly baseline visits before disaster) Increase in weekly, hypertensive kidney disease-related visits (one week after disaster): + 8 cases (reference: weekly baseline visits before disaster) Increase in weekly, myocardial infarction-related visits (one week after disaster): + 8 cases (reference: weekly baseline visits before disaster)
New Jersey, Superstorm Sandy (2012)^45^	ED patients with a primary diagnosis of diabetes	Increase in acute care-related ED visits (week of disaster): + 84%; 95% CI, 1.12–3.04; *P* = .01 (reference: control week from year before disaster)
Louisiana, Mississippi, Texas, and Alabama, Hurricanes Katrina and Rita (2005)^46^	Medicare enrollees with diabetes	Increase in total ED visits (three years after disaster): + 21,583 visits; 95% CI, 11,676–31,490 (reference: unaffected control group)
**Individuals with Mental Health Symptoms**		
New York City, Superstorm Sandy (2005)^47^	ED patients presenting with psychiatric concerns	Increase in psychiatry-related ED visits (year after disaster): Close to + 32%; *P* < .001 (reference: year before disaster)

*ED*, emergency department.
